# orthoSynAssign: refine orthogroups using synteny information

**DOI:** 10.64898/2026.08.10.744007

**Published:** 2026-08-11

**Authors:** Cheng-Hung Tsai, Carolina G. Piña Páez, Jason E. Stajich

## Abstract

Accurately identifying orthogroups is crucial for precise phylogenetic reconstruction, but clustering-based methods often generate complex, many-to-many orthogroups that include confounding paralogs. Incorporating synteny offers a robust strategy to refine these clusters into high-granularity, single-copy orthologs. We introduce orthoSynAssign, a user-friendly, high-performance rewrite of the orthogroup refinement tool OrthoRefine, combining an intuitive Python interface with a core computing engine written in Rust. This hybrid architecture ensures straightforward installation, seamless data parsing, and exceptional computational efficiency. Evaluated against the Yeast Gene Order Browser (YGOB) dataset, orthoSynAssign demonstrated outstanding performance, substantially elevating the Area Under the Precision-Recall Curve. Furthermore, multi-threading benchmarks across 193 Eurotiomycetes genomes confirmed strong scalability, drastically reducing execution runtime while maintaining a strictly bounded, thread-independent memory footprint. Ultimately, orthoSynAssign provides a reliable and scalable framework for high-throughput phylogenomic workflows.

## Full Text Availability

The license terms selected by the author(s) for this preprint version do not permit archiving in PMC. The full text is available from the preprint server.

